# Prevalence of sexual harassment and its association with aspects self-reported health: A cross-sectional study in Sweden

**DOI:** 10.1177/14034948241248684

**Published:** 2024-05-09

**Authors:** Malin Hagland, Gunnar J. Brandén, Kyriaki Kosidou, Anna M. Nielsen

**Affiliations:** 1 Department of Global Public Health, Karolinska Institute, Sweden; 2 Center for Epidemiology and Community Medicine, Region Stockholm, Sweden

**Keywords:** Sexual harassment, sexual identity, mental health, depression, anxiety

## Abstract

**Aims:**

Sexual harassment is common and may have serious consequences for well-being and health. We investigated the prevalence of sexual harassment in the general population of Stockholm County across socio-demographic groups and sexual identity, and its association with self-reported health.

**Methods:**

Experiences of sexual harassment in the previous 12 months and its associations with self-reported health (depression, anxiety, suicidal ideation) were assessed in 2021/2022 among a random sample of 22,890 residents in Stockholm County aged 16 and older. Analyses were done using descriptive statistics and multivariate logistic regression (odds ratios and 95% confidence intervals (CI)). Calibration weights were used to estimate population-representative rates with 95% CI.

**Results:**

The highest weighted prevalence was observed among 16- to 24-year-olds (18.9%, 95% CI 16.9%–20.9%). Women (9.5%; 95% CI 8.8%–10.1%) reported a higher prevalence than men (2.9%; 95% CI 2.5%–3.3%). Compared to heterosexual people, bisexual and homosexual people reported a higher prevalence of sexual harassment. No significant difference was observed related to country of birth. People who were students, unemployed or on sick leave were more exposed than employed people, although this was not significant when adjusted for age and registered sex. Experiences of sexual harassment was associated with higher odds of all aspects of self-reported health.

**Conclusions:**

**Experiences of sexual harassment in the general population are common and associated with adverse self-reported health. There is a need for enhanced efforts to prevent sexual harassment in the general population and to empower specific risk groups such as women and sexual minorities.**

## Introduction

Sexual harassment (SH) is a public health problem that gained widespread recognition during the #MeToo movement in 2017 [1]. According to the World Health Organization (WHO), SH falls under the concept of sexual violence, which is defined as: ‘any sexual act, attempt to obtain a sexual act, unwanted sexual comments or advances, or acts to traffic, or otherwise directed, against a person’s sexuality using coercion’ [2]. Although there is no general agreement, SH is commonly defined as unwanted conduct that has the purpose or effect of being intimidating, hostile, degrading, humiliating or offensive [3]. Definitions of SH among victims vary, but examples include deeming jokes, comments about appearances, sexual propositions and sexual favours [3–5].

The European Union Agency for Fundamental Rights (FRA) estimated that of all European women, 13%–21% have experienced SH in the previous 12 months and 45%–55% at sometime during their lifetime [6]. In a national survey, 42% of Swedish women and 9% of men aged 16–84 reported experience of SH during their lifetime [7]. Furthermore, studies from Sweden and elsewhere have indicated differences in prevalence across socio-demographic groups and sexual identities. For instance, young people were more exposed compared to older people, women were more exposed compared to men, and non-heterosexual people more exposed compared to heterosexual people [3,5,7–9].

Associations between experiences of SH and poor mental health have previously been shown [4,5,8,10–12], including depression, anxiety, post-traumatic stress disorder (PTSD) and suicide – that is, conditions of public health relevance due to their impact on well-being and mortality. A Swedish study revealed SH to be associated with a doubled risk for adverse mental health, with this risk being significantly greater among women [8]. Given the high prevalence of depression and anxiety in Swedish society [13], and the severity of suicidal ideation, we deemed it important to include these outcomes in our analysis.

Most previous studies have focused on specific settings such as schools, universities or workplaces [5,14,15] or have had small study population [6,9], which limits the generalisability of the findings to the general population. Furthermore, lifetime prevalence [7,9] introduces recall bias and is less reliable compared to prevalence during the past 12 months. Updated SH prevalence estimates based on data from a large population-based study and its potential associations with health are lacking. This study will serve as baseline for prevalence studies in the urban Swedish context, as well as a basis for public health interventions to prevent SH.

### Aim

The aims of this study were to investigate the prevalence of SH in the previous 12 months across different socio-demographic groups and sexual identities and to examine the associations of SH with aspects of self-reported health.

## Methods

### Study population and setting

This study was conducted among the general population of Stockholm County. Stockholm is the capital of Sweden, with 2.5 million residents, among whom 1.6 million live in the urban area [16]. Sweden’s gender equality index was 82.2/100 in 2023, which is the highest in the European Union [17].

This cross-sectional study used population-based data from a survey conducted in Stockholm County between September 2021 and May 2022 [13]. The sampling frame consisted of all people aged 16 or older in the Swedish National Population Register residing in Stockholm County in 2021. In total, 47,855 individuals were randomly selected in an area-stratified manner (based on residency in 39 municipalities or urban districts). The survey used web-based or postal questionnaires distributed via the participants’ addresses. In total, 23,066 individuals responded to the survey, giving a response rate of 48%. Among the respondents, 155 individuals did not reply to the question about SH and were excluded, resulting in a final analytical sample of 22,911 individuals. The questionnaires comprised 86 questions, including socio-demographic and health parameters. The respondents were linked to several administrative and health-care registers through the Swedish personal identification number, which was removed prior to analysis. Ethical approval was granted by the Stockholm Regional Ethical Review Board (dnr. 2022-02626-01).

### Co-variates

#### Sexual harassment

SH was assessed by the question: ‘In the previous 12 months, have you been sexually harassed online or in person? For example, unwanted letters, text messages, e-mails, phone calls or pictures with sexual content, unwanted sexual suggestions or comments, sexual defamation or flashers’. The response alternatives were: ‘Yes, on multiple occasions’, ‘Yes, on a single occasion’ and ‘No’. Responses were dichotomised into ‘Yes, any experience within the previous 12 months’ and ‘No’.

#### Aspects of self-reported health

Symptoms of depression were assessed with the well-established Patient Health Questionnaire (PHQ-2). The PHQ-2 is a modified version of the PHQ-9 and has been validated to screen for depression [18]. Based on previous literature, a total score of three or more was used as a cut-off for symptoms of depression. Symptoms of anxiety were assessed using the validated General Anxiety Disorder 2 (GAD-2) instrument, a modified version of the GAD-7. We used a cut-off score of three or more to denote symptoms of anxiety in accordance with previous studies [18]. Suicidal ideation was assessed with the question: ‘Have you ever at any point in time seriously considered committing suicide, perhaps even planned it?’ The response alternatives were: ‘Never’, ‘Yes, more than a year ago’, ‘Yes, within the previous 12 months’ or ‘Yes, within the past week’. The responses were dichotomised into ‘Suicidal ideation within the previous 12 months’ (including past week) and ‘No suicidal ideation within the previous 12 months’ (including suicidal ideation more than a year ago).

#### Sexual identity and socio-demographic characteristics

Sexual identity was assessed by the item ‘How would you define your sexual identity?’ The response alternatives were: ‘Heterosexual’, ‘Homosexual’, ‘Bisexual’ or ‘None of the above’. Self-reported employment status was obtained via the questionnaire and was categorised as ‘Employed’, ‘Unemployed or on sick leave’, ‘Student’, ‘Retired’ and ‘Other’. The National Population Registry provided data on registered sex, age (categorised as 16–24, 25–34, 35–44, 45–55, 54–65 and 65+ years old) and country of birth (coded as Sweden, Global North and Global South) [19].

### Statistical analysis

Calibration weights were used in the demographic analyses to adjust for the stratified random sample design and systematic differences in non-response based on auxiliary information [20]. Calibration weights were not applied to the logistic regression models, as this would decrease the accuracy of the estimations. Auxiliary information included age, registered sex, educational level, marital status and area of residence. All analyses were performed using the statistical software Stata v17 (StataCorp LLC, College Station, TX), and statistical significance was tested at *p*⩽0.05.

The prevalence rates of SH overall and by registered sex, age groups, sexual identity, employment status and country of birth were reported using weighted univariate statistics (with 95% confidence intervals (CI)). We used logistic regression adjusted for registered sex and age group to estimate odds ratios (ORs) with 95% CI for the association between SH and given variables.

We examined the associations between SH and self-reported health using logistic regression. Two models were specified: an unadjusted model and a model adjusted for the possible confounders (age group, registered sex and sexual identity). Regression analyses were stratified by registered sex with an unadjusted model and a model adjusted for age groups and sexual identity. The confounding variables were selected based on previously observed associations with SH and/or the selected aspects of self-reported health [8,12,14].

## Results

Table I shows the characteristics of study participants by experiences of SH within the previous 12 months. In total, 50.5% of the study participants were female, 71.3% were born in Sweden and 84.6% were heterosexual. The self-reported prevalence rates of adverse mental health were 11.1% for anxiety, 11.5% for depression and 3.9% for suicidal ideations.

**Table I.** Characteristics of study participants by experiences of sexual harassment.

		No sexual harassment			Experienced sexual harassment			Total		
		Col. % (weighted)	Col. % (unweighted)	*n*	Col. % (weighted)	Col. % (unweighted)	*n*	Col. % (weighted)	Col. % (unweighted)	*n*
Total		100.0	100.0	21661	100.0	100.0	1250	100.0	100.0	22911
Registered sex	Men	51.2	47.6	10307	23.2	21.3	266	49.5	46.1	10573
	Women	48.8	52.4	11354	76.8	78.7	984	50.5	53.9	12338
Age groups (years)	65–100	22.3	29.8	6459	6.9	9.7	121	21.4	28.7	6580
	55–64	16.3	18.0	3907	8.5	11.1	139	15.8	17.7	4046
	45–54	16.3	18.2	3948	10.7	13.3	166	16.0	18.0	4114
	35–44	18.9	15.4	3340	15.9	15.3	191	18.7	15.4	3531
	25–34	15.8	11.7	2543	21.7	20.8	260	16.2	12.2	2803
	16–24	10.4	6.8	1464	36.3	29.8	373	12.0	8.0	1837
	Missing									
Sexual identity	Heterosexual	85.2	88.0	19053	75.8	77.8	972	84.6	87.4	20025
	Bisexual	2.5	2.0	438	11.2	11.7	146	3.0	2.5	584
	Homosexual	1.8	1.5	330	3.3	2.9	36	1.9	1.6	366
	None of the above	8.3	6.7	1443	7.3	5.7	71	8.3	6.6	1514
	Missing	2.1	1.8	397	2.5	2.0	25	2.1	1.8	422
Employment status	Employed	60.7	58.6	12694	54.3	55.7	696	60.3	58.4	13390
	Unemployed or on sick leave	5.0	4.3	925	5.9	6.2	77	5.0	4.4	1002
	Student	9.9	6.8	1470	29.4	25.4	317	11.2	7.8	1787
	Retired	19.3	26.0	5625	5.5	7.9	99	18.5	25.0	5724
	Other	3.1	2.7	593	2.4	2.8	35	3.1	2.7	628
	Missing	1.9	1.6	354	2.5	2.1	26	1.9	1.7	380
Country of birth	Sweden	71.1	78.5	17013	74.3	80.1	1001	71.3	78.6	18014
	Global North	13.6	11.2	2433	9.9	8.9	111	13.4	11.1	2544
	Global South	15.4	10.2	2215	15.8	11.0	138	15.4	10.3	2353
	Missing									
Anxiety	No anxiety	87.7	88.9	19267	73.5	76.3	954	86.8	88.3	20221
	Anxiety	10.1	8.9	1935	24.9	22.6	282	11.1	9.7	2217
	Missing	2.2	2.1	459	1.6	1.1	14	2.1	2.1	473
Depression	No depression	87.1	88.5	19163	76.3	78.1	976	86.4	87.9	20139
	Possible depression	10.8	9.4	2039	22.5	20.9	261	11.5	10.0	2300
	Missing	2.1	2.1	459	1.2	1.0	13	2.1	2.1	472
Suicidal ideation	No suicidal ideation	96.0	96.3	20864	87.2	88.4	1105	95.4	95.9	21969
	Suicidal ideation	3.4	3.0	653	12.0	11.0	137	3.9	3.4	790
	Missing	0.7	0.7	144	0.8	0.6	8	0.7	0.7	152

Sexual harassment was assessed within the previous 12 months among the general population of Stockholm County aged 16–100 years in 2021–2022. *N*=22,911.

Table II shows crude cases and weighted prevalence of SH among different socio-demographic groups and by sexual identity, stratified by registered sex and in total. The associations adjusted for sex and age groups are also presented in Table II. The overall weighted prevalence within the previous 12 months was estimated at 6.2% (95% CI 5.9%–6.6%). One third (33.4%; 95% CI 30.1%–36.8%) of women between 16 and 24 years of age had experienced SH. The prevalence of SH was generally higher among women compared to men and higher among younger compared to older individuals. Bisexual people (23.1%; 95% CI 19.3%–27.0%) more commonly reported experiences of SH compared to heterosexuals (5.6%; 95% CI 5.2%–6.0%). Among employment groups, students reported one of the highest prevalence for both sexes, although the difference was not statistically significant when adjusted for age groups and sex. Compared to bisexual men (28.2%; 95% CI 23.3%–33.2%), bisexual women reported a higher prevalence of SH (11.5%; 95% CI 6.2%–16.9%). Exposure to SH was also more common among female students compared to male students. The unweighted prevalence was similar to the weighted prevalence.

**Table II.** Weighted prevalence of sexual harassment in the previous 12 months and its adjusted association with socio-demographic groups and sexual identity for age groups and registered sex.

		Men		Women		Total		OR adjusted for age groups and registered sex
		Cases (*n*)	Weighted prevalence % (CI)	Cases (*n*)	Weighted prevalence % (CI)	Cases (*n*)	Weighted prevalence % (CI)	
Total		266	2.9 (2.5–3.3)	984	9.5 (8.8–10.1)	1250	6.2 (5.9–6.6)	
Age groups (years)	65–100	45	1.6 (1.1–2.2)	76	2.3 (1.8–2.9)	121	2.0 (1.6–2.4)	
	55–64	57	2.7 (1.9–3.5)	82	4.0 (3.0–4.9)	139	3.3 (2.7–4.0)	
	45–54	40	2.2 (1.4–3.0)	126	6.2 (5.0–7.4)	166	4.2 (3.5–4.9)	
	35–44	37	2.8 (1.8–3.8)	154	7.8 (6.5–9.1)	191	5.3 (4.5–6.2)	
	25–34	39	3.4 (2.2–4.5)	221	13.3 (11.5–15.1)	260	8.4 (7.3–9.5)	
	16–24	48	5.7 (3.9–7.5)	325	33.4 (30.1–36.8)	373	18.9 (16.9–20.9)	
Sexual identity	Heterosexual	193	2.3 (1.9–2.7)	779	8.9 (8.2–9.6)	972	5.6 (5.2–6.0)	1.00
	Bisexual	22	11.5 (6.2–16.9)	124	28.2 (23.3–33.2)	146	23.1 (19.3–27.0)	2.28*** (1.75–2.96)
	Homosexual	16	9.5 (4.7–14.2)	20	12.5 (6.8–18.2)	36	10.6 (7.0–14.3)	1.84** (1.18–2.86)
	None of the above	26	5.1 (3.0–7.3)	45	5.7 (3.9–7.6)	71	5.5 (4.1–6.9)	0.95 (0.70–1.27)
Employment status	Employed	154	2.8 (2.3–3.3)	542	8.6 (7.8–9.4)	696	5.6 (5.2–6.1)	1.00
	Unemployed or on sick leave	14	3.4 (1.4–5.4)	63	10.8 (7.9–13.7)	77	7.3 (5.5–9.1)	1.24 (0.92–1.67)
	Student	42	5.0 (3.3–6.7)	275	26.8 (23.7–29.9)	317	16.5 (14.6–18.4)	1.19 (0.94–1.51)
	Retired	36	1.5 (0.9–2.1)	63	2.1 (1.5–2.7)	99	1.8 (1.4–2.3)	0.48** (0.30–0.78)
	Other	12	4.0 (1.5–6.5)	23	5.5 (3.0–8.0)	35	4.8 (3.0–6.6)	0.70 (0.46–1.05)
Country of birth	Sweden	193	2.6 (2.2–3.1)	808	10.3 (9.5–11.1)	1001	6.5 (6.1–7.0)	
	Global North	22	2.3 (1.2–3.4)	89	6.7 (5.2–8.2)	111	4.6 (3.7–5.6)	0.88 (0.70–1.12)
	Global South	51	4.7 (3.3–6.1)	87	8.2 (6.4–10.0)	138	6.4 (5.3–7.6)	1.07 (0.86–1.33)

Estimated odds ratios (ORs) adjusted for age groups and registered sex, with 95% confidence intervals (CI). Sexual harassment was assessed within the previous 12 months among the general population of Stockholm County aged 16–100 years in 2021–2022. *N*=22,911. **p<0.01; ***p<0.001.

Table III presents the associations between SH and aspects of self-reported health for men, women and in total. Experiences of SH were significantly associated with depression, anxiety and suicidal ideation after adjusting for age groups, sex and sexual identity. The association between all health outcomes and SH was slightly higher among women compared to men, although the difference was not significant (interaction not shown). The strongest association was observed between experience of SH and suicidal ideation (total adjusted OR=2.62; 95% CI 2.11–3.25). Experience of SH increased the likelihood for symptoms of depression by twofold among women (OR=2.00; 95% CI 1.67–2.40) and almost twofold among men (OR=1.71; 95% CI 1.33–2.42). Both men and women exposed to SH were almost twice as likely to experience symptoms of anxiety compared to those not exposed to SH.

**Table III.** Association between prevalence of sexual harassment and aspects of self-reported health.

	Unadjusted		Adjusted	
	OR	95% CI	OR	95% CI
*Total*				
Depression	2.51***	(2.18–2.90)	1.94***,^a^	(1.66–2.27)
Anxiety	2.94***	(2.56–3.39)	1.88***,^a^	(1.61–2.19)
Suicidal ideation	3.96***	(3.26–4.81)	2.62***,^a^	(2.11–3.25)
*Women*				
Depression	2.55***	(2.17–3.00)	2.00***,^b^	(1.67–2.40)
Anxiety	2.76***	(2.36–3.23)	1.84***,^b^	(1.55–2.19)
Suicidal ideation	4.23***	(3.39–5.29)	2.69***,^b^	(2.09–3.46)
*Men*				
Depression	2.04***	(1.46–2.84)	1.71**,^b^	(1.21–2.42)
Anxiety	2.17***	(1.53–3.09)	1.74**,^b^	(1.20–2.52)
Suicidal ideation	2.93***	(1.87–4.60)	2.15**,^b^	(1.33–3.48)

Association assessed as odds ratio (OR) with 95% confidence interval (CI), using unweighted data. Unexposed individuals were used as reference categories for all health outcomes. Sexual harassment was assessed within the previous 12 months among the general population of Stockholm County aged 16–100 years in 2021–2022. *N*=22,911.

a Adjusted for age groups, registered sex and sexual identity.

b Adjusted for age groups and sexual identity.

** *p*⩽0.01; ***p⩽0.001.

## Discussion

SH is a prevalent problem in Stockholm County, with approximately one third of young women exposed within the previous 12 months. SH is more common among bisexual and homosexual people compared to heterosexual people. Furthermore, our results illustrate a markedly strong association between experiences of SH and anxiety, depression and suicidal ideation.

### Gender, sexual identity and power

In accordance with other studies, young women were unproportionally exposed to SH [3,5,7,15]. When interpreting the large difference in prevalence between the sexes, it is important to consider gender norms and patriarchal power structures. SH is considered part of the continuum of sexual violence, with hierarchal power relations at its core [2,3,21]. SH can be viewed as means to facilitate systemic patriarchal power asymmetry, which contributes to the higher risk among women [7,8,10]. Nevertheless, gender normative limitations are important to consider – that is, masculinity norms might restrict men from reporting and identifying certain experiences as SH [3,22]. Hence, prevalence among men might be underestimated but is unlikely to explain the gendered difference fully.

Previous studies among adolescents suggest that SH serves as a means to reinforce heteronormative masculinity rather than being an expression of sexual desire [23,24]. SH is thus understood as a way to facilitate heteronormative power asymmetry. This can also be applied to an adult population as systematic oppression is believed to be similar, and sexual minority adults experience a higher prevalence of SH, in all age groups [3,5,7]. Sexual minorities must therefore be prioritised in preventive efforts – for example, by avoiding a heteronormative perspective [23,24].

### Association with aspects of self-reported health

As reported elsewhere, we found significant associations between explored aspects of self-rated health and experiences of SH for both men and women [10–12,14]. The associations between SH and adverse self-rated health were slightly higher among women compared to men, which is in line with previous research from Sweden [8]. Despite limited evidence regarding the temporal association, previous studies suggest that experiences of SH negatively impact mental health, such as depression, anxiety and suicide [8,11,12]. For example, a Swedish study showed a prospective association between workplace SH and committing suicide [11], which is in line with findings in our study. However, another study among adolescents in Sweden found mixed results regarding the temporal associations between SH and depression, depending on gender and different acts of SH [12]. The possible bidirectional association between SH and adverse health does not diminish the need to prevent SH, as this could both prevent adverse health in exposed individuals and/or protect vulnerable groups from exposure to SH.

### Comparison with previous studies

Few studies have investigated the prevalence of SH in the previous 12 months among a general population during recent years in Sweden [6,8,15]. The prevalence of SH observed in our study was slightly higher compared to national studies [8,15] but lower compared to a European study [6]. Comparing prevalence of SH is complicated due to differences in assessment methods, contextual factors and conceptual understanding of SH among and between populations [5,6,21,25,26]. For example, the European study used excessive assessment – that is, asking about specific situations of SH compared to the single-item question in our study – which is known to increase the obtained prevalence [6,25,26]. Lifetime prevalence is not comparable to the one-year prevalence observed in this study [7,9]. The limitations of comparing prevalence across studies highlight the potential of the current study to function as a baseline for the temporal assessment of prevalence.

Observed differences across socio-demographic groups and sexual identity are in line with previous findings [3–5,7,8]. In our study, retired people were less exposed compared to employed individuals. In addition, students, unemployed individuals and individuals on sick leave had a higher prevalence of SH compared to employed individuals, although the associations were not significant when adjusted for sex and age groups. Previous studies in university settings confirm SH to be a prevalent problem for students and indicate specific risks associated with being a student apart from age [5,22]. The relatively high prevalence rates among unemployed people and people on sick leave highlight the need to prevent SH outside work and university settings.

The prevalence of SH was not significantly different between people born in Sweden compared to people born outside Sweden, which is in line with a previous Swedish study [8]. Findings among university students indicated an increased risk for exposure among white students compared to ethnic minorities, although these results should be interpreted with caution due to limitations of methods to capture both gender and racial harassment [5].

### Limitations

Our study has many strengths, including a large and representative population-based sample with extensive information on health and socio-demographic factors. However, one of the main limitations in the current study is the assessment of SH by a singular item. This has potentially limited our ability to capture all SH events, and consequently the prevalence might be underestimated. Previous studies have emphasised the need to include several questions on specific acts of SH to promote a uniform understanding of the term and to increase participants’ tendency to identify experiences as SH [25,26]. However, multiple examples of SH were provided in our survey, which probably increase the sensitivity of the assessment. The scope of the survey narrowed assessment of SH to a single-item question, which limited a detailed understanding of setting, context and perpetrator. Thus, we were not able to assess variations in types of SH, which is known to differ [8,15]. In addition, we were not able to investigate online SH specifically, known to be common among young people [21,24]. We were not able to assess the relation between SH and other forms of sexual violence suggested by previous studies [2]. This overlap and how that might affect the association with self-rated health are therefore not known.

Yet another limitation is the cross-sectional study design, which limits causal interpretation of the association between self-reported health, socio-demographic factors and SH. We cannot know whether adverse self-reported health preceded SH.

Furthermore, voluntary public health surveys inherit selection bias, as the non-respondents are likely to differ from respondents in terms of socio-economic and health factors [26,27]. This is partly counterbalanced by the calibration weights, although it is still important to consider.

### Implications

Despite the limitations of the current study design and difficulties assessing SH, our results provide a relevant baseline for future monitoring of the problem and provide implications for future work. The high prevalence among young women emphasises the public health relevance of SH and the need for preventive measures. Furthermore, the results shed light on the importance of working broadly to prevent systematic power asymmetry, even in a country with gender equality as high as seen in Sweden. Interventions should focus on preventing perpetration and empowering risk groups to identify and report SH. As indicated in previous literature, the intersectional perspective should be applied to understand exposure to SH fully and to develop appropriate interventions, as gender power can intersect with other hierarchical structures [3–5]. The associations between SH and adverse self-reported health can have policy implications for prevention and care, despite causal direction. For example, screening for SH should be considered during psychological assessment and treatment.

Given the large and representative sample in this study, the results could be generalisable to urban settings in high-income countries with similar features as Stockholm County. Furthermore, this study plays an essential role in providing a baseline for future follow-up studies on SH in Stockholm County and thus constitutes a base for prospective associations. Longitudinal follow-up will allow indirect evaluation of targeted public health interventions, such as changes in school curriculum, legislative changes, awareness-raising interventions and active bystander interventions.

## Conclusions

In conclusion, our results highlight SH as a prevalent public health problem associated with adverse health. Women and people of sexual minorities are particularly vulnerable to SH, which should be considered when developing preventive initiatives. Our results, emphasise the importance of maintaining the public health focus on SH and sustained preventive efforts.

It is further important to conduct more in-depth analyses of SH to gain a detailed understanding of where, how, when, and by whom the harassment occurs, especially in relation to online arenas. Moving forward, longitudinal analyses are needed to assess a causal relationship between SH and aspects of self-reported health. The overlap between SH and other forms of sexual violence and its implications for health is important to investigate further. Public health researchers and practitioners have an important role to play in implementing preventative strategies for SH.
